# IgLON4 Regulates Myogenesis via Promoting Cell Adhesion and Maintaining Myotube Orientation

**DOI:** 10.3390/cells11203265

**Published:** 2022-10-17

**Authors:** Jeong Ho Lim, Khurshid Ahmad, Hee Jin Chun, Ye Chan Hwang, Afsha Fatima Qadri, Shahid Ali, Syed Sayeed Ahmad, Sibhghatulla Shaikh, Jungseok Choi, Jihoe Kim, Jun-O Jin, Myunghee Kim, Sung Soo Han, Inho Choi, Eun Ju Lee

**Affiliations:** 1 Department of Medical Biotechnology, Yeungnam University, Gyeongsan 38541, Korea; 2 Research Institute of Cell Culture, Yeungnam University, Gyeongsan 38541, Korea; 3 Department of Animal Science, Chungbuk National University, Cheongju 28644, Korea; 4 Department of Food Science and Technology, Yeungnam University, Gyeongsan 38541, Korea; 5 School of Chemical Engineering, Yeungnam University, Gyeongsan 38541, Korea

**Keywords:** skeletal muscle, IgLON4, lipid raft, differentiation, myotube orientation, myotube alignment

## Abstract

Immunoglobulin-like cell adhesion molecule (IgLON4) is a glycosylphosphatidylinositol-anchored membrane protein that has been associated with neuronal growth and connectivity, and its deficiency has been linked to increased fat mass and low muscle mass. Adequate information on IgLON4 is lacking, especially in the context of skeletal muscle. In this study, we report that IgLON4 is profusely expressed in mouse muscles and is intensely localized on the cell membrane. IgLON4 expression was elevated in CTX-injected mouse muscles, which confirmed its role during muscle regeneration, and was abundantly expressed at high concentrations at cell-to-cell adhesion and interaction sites during muscle differentiation. IgLON4 inhibition profoundly affected myotube alignment, and directional analysis confirmed this effect. Additionally, results demonstrating a link between IgLON4 and lipid rafts during myogenic differentiation suggest that IgLON4 promotes differentiation by increasing lipid raft accumulation. These findings support the notion that a well-aligned environment promotes myoblast differentiation. Collectively, IgLON4 plays a novel role in myogenesis and regeneration, facilitates myotube orientation, and is involved in lipid raft accumulation.

## 1. Introduction

Skeletal muscles (SMs) are attached to bones by tendons and enable movement by transmitting their contractions and relaxations to the skeleton. The foremost function of SM is to convert chemical energy into physical and thermal energy to facilitate motility and maintain posture and body temperature [1]. SM is made of multinucleated myofibers and resident stem cells, namely muscle satellite (stem) cells (MSCs), which dynamically govern myofiber growth and progression via myogenic regulatory factors [2,3]. Myogenesis is a highly regulated process that involves the differentiation of multipotent mesenchymal cells into myoblasts, which then differentiate and fuse to produce myofibers.

Extracellular matrix (ECM) components, membrane proteins, and transmembrane receptors directly or indirectly participate in muscle development, growth, repair, contraction, and force transmission [4,5]. Earlier studies by our group and others have demonstrated that several ECM components, such as fibromodulin (FMOD) [6,7,8], matrix gla protein (MGP) [9], fibronectin [10,11], and dermatopontin (DPT) [12], play vital roles in the regulation of myogenesis. FMOD is widely recognized for its ability to attach directly to collagen (a major component of ECM) and its regulation of fibrillogenesis [13]. Furthermore, FMOD is involved in the development of SM ECM, has a substantial effect on the expressions of myogenic marker genes (myoblast determination protein (MYOD), myogenin (MYOG), and myosin light chain 2 (MYL2)), regulates myogenesis, and is actively involved in the recruitment of MSCs to injured muscle sites. Genes that were differentially expressed in FMOD knockdown and normal C2C12 myoblast differentiation were identified using microarray analysis, and immunoglobulin-like cell adhesion molecule 5 (IgLON5) was found to be consistently and significantly downregulated during differentiation in the knockdown of FMOD [6,7]. Little research has been performed on the roles of IgLON family members in the context of SM.

The role of IgLON5 in myogenesis and muscle regeneration was recently examined, and it was suggested that IgLON5 is implicated in muscle regeneration and linked with a variety of genes, particularly ECM genes, and plays roles in C2C12 cell adhesion and differentiation [14]. The IgLON family is a subfamily of the immunoglobulin superfamily (IgSF), members of which have an immunoglobulin (Ig) domain and share several fundamental characteristics (e.g., cell-membrane binding, cell-to-cell recognition, and adhesion). The five members of the IgLON family are: IgLON1-opioid binding cell adhesion molecule (OPCML), IgLON2-neurotrimin (NTM), IgLON3-limbic system-associated membrane protein (LSAMP), IgLON4-neuronal growth regulator 1 (NEGR1), and IgLON5. IgLONs control neurite outgrowth, dendritic arborization, and synapse formation [15].

IgLONs also possess a glycosylphosphatidylinositol (GPI) entity, which anchors to the cell membrane, and three-conserved Ig-like C2 domains, and are primarily found in lipid rafts. Lipid rafts float like rafts on cell membranes, and are defined by GPI-anchored proteins like those found in IgLONs [16]. These specialized membrane microdomains are rich in sphingolipids and cholesterol, and the proteins found mainly in ‘lipid rafts’ share properties with GPI-anchored proteins, doubly acylated proteins, cholesterol-linked proteins, and palmitoylated proteins, and perform functions such as membrane sorting, receptor trafficking, signal transduction, and cell adhesion [16,17]. Therefore, to understand how IgLONs function, an understanding of how IgLONs interact with the lipid rafts where they are localized in cell membranes is required.

IgLON4 has a critical function during neurodevelopment. In a study that confirmed increased expression of primary astrocyte neurons during neurite formation in vitro, it was observed that IgLON4 protein increased cell-to-cell recognition and neurite outgrowth [18]. Kim et al. explored the role of IgLON4 in obesity and various cancers and found its expression was frequently decreased in a range of human cancers, implying that IgLON4 modulates cell adhesion, which is essential for cell growth and cell-to-cell communication [19]. Furthermore, a recent study using an IgLON4 knockout mouse model showed that IgLON4 caused mice to store fat but lose muscle [20].

Due to the inadequacy of information on IgLON4, particularly in the context of SM, and its possible role in lipid raft-associated mechanisms, we investigate its roles in SM myogenesis, cell adhesion, and myotube orientation. In addition, we investigated the functions of IgLON4 in lipid rafts during differentiation.

## 2. Materials and Methods

### 2.1. Cell Culture

#### 2.1.1. C2C12 Myoblast Cells

Murine C2C12 myoblast cells (Korean Cell Line Bank, Seoul, Korea) were cultured in growth media DMEM (Dulbecco’s Modified Eagle’s Medium) supplemented with 10% FBS (Fetal Bovine Serum) and 1% P/S (penicillin/streptomycin; all were purchased from Hyclone, South Logan, UT, USA) in a humidified 5% CO_2_ incubator at 37 °C. When cells achieved 90% confluence, growth media was switched to differentiation media (DMEM supplemented with 2% FBS and 1% P/S) and cultured for 0, 2, 4, or 6 days with daily media changes. The same conditions were used to culture cells in striped culture dishes (Curi Bio Inc, Seattle, WA, USA).

#### 2.1.2. Mouse MSC Cultures

Mouse MSCs were cultured in Ham’s F-10 growth medium (F-10 Nutrient Mixture, Hyclone, South Logan, UT, USA) supplemented with 20% FBS, 1% P/S, and 5 ng/mL of fibroblast growth factor 2 (FGF2, Miltenyi Biotec, Auburn, CA, USA) in a humidified 5% CO_2_ incubator at 37 °C. When cells achieved 90% confluence, the growth media was switched to a differentiation medium (DMEM supplemented with 2% FBS and 1% P/S) and cultured for 0, 2, or 4 days with daily media changes.

### 2.2. Mouse MSC Isolation

Mouse gastrocnemius (gas) muscles were collected from C57BL male mice (six weeks), minced, digested with 1% pronase (Roche, Mannheim, Germany) for 1 h at 37 °C, and centrifuged at 1000× *g* for 3 min. The digested tissue phase was passed through a 100 mm cell vacuum strainer (Millipore, Darmstadt, Germany), and the filtrate was centrifuged at 1000× *g* for 5 min. Pellets were suspended in Ham’s F-10 + 20% FBS + 1% P/S + 5 ng/mL FGF2, seeded on collagen-coated plates (Corning, Brooklyn, NY, USA), and incubated in a humidified 5% CO_2_ incubator at 37 °C. MSC purity was confirmed by PAX7 and MYOD immunocytochemistry.

### 2.3. Neutralization of IgLON4

C2C12 cells grown to 90% confluence were replaced with differentiation medium and treated with 3 μg/mL IgLON4 antibody (Santa Cruz Biotechnology, Santa Cruz, CA, USA) at the same time. Then, the cells were cultured on the second day and the fourth day.

### 2.4. Gene Knockdown

Cells were transfected with IgLON4 (1 ng), IgLON5 (1 ng), or IgLON4 (1 ng) and IgLON5 (1 ng) shRNA, or a scrambled vector (Santa Cruz Biotechnology, Santa Cruz, CA, USA). Knockdown and selection were performed as previously described [14]. The shRNA sequence information is provided in Supplementary Table S1.

### 2.5. Cell Adhesion and Proliferation Assays

Equal numbers of Wt (wild type), IgLON4_kd_ (IgLON4 knockdown), IgLON5_kd_ (IgLON5 knockdown), and Db_kd_ (IgLON4 and 5 double knockdown) cells were cultured in growth medium for 3 h for the adhesion study or 3 days for the proliferation study. Cell proliferation was performed by MTS assays [21]. The method was performed as follows. The cells were then incubated in CellTiter 96^®^ AQueous One Solution Reagent (Promega, Madison, WI, USA) for 1 hr in a humidified 5% CO_2_ incubator at 37 °C. Absorbances were measured at 490 nm using a microplate reader (Biotek Synergy H1, Winooski, VT, USA). Proliferation rates were normalized with respect to adherent Wt, IgLON4_kd_, IgLON5_kd_, or Db_kd_ cells cultured for 3 h.

### 2.6. Centrifugation-Based Assessment of Adhesion Force

Wt, IgLON4_kd_, IgLON5_kd,_ or Db_kd_ cells were cultured in growth media until they reached ~30% or 100% confluency or in a differentiation medium for 4 days. Then, cell culture plates were filled with growth or differentiation media and sealed with microplate sealing tape. Cell culture plates were then inverted and centrifuged at three different ‘g’ values (100× *g* for 30% confluent cells, 500× *g* for 100% confluent cells, or 1000× *g* for cells differentiated for 4 days) in a centrifuge with a swinging bucket rotor for 5 min. MTS assays were used to measure cell adhesion after centrifugation, as described above [21].

### 2.7. The Animal Experiments

The C57BL/6 male mice (Daehan Biolink, Dae-Jeon, Korea) were maintained in a temperature-controlled room under a 12 h light/12 h dark cycle with free access to water and a normal diet (4.0% (w/w) total fat; Rodent NIH-31 Open Formula Auto; Zeigler Bros., Inc., Gardners, PA, USA). Mice were anesthetized with avertin before being injected with 100 µL of 10 mM cardiotoxin (CTX) or PBS (control) into the left or right gas muscles, respectively. Gas muscles were excised 7 days after injection, fixed, and stored at −80 °C for further analysis. All experiments were conducted in accordance with the guidelines issued by the Institutional Animal Care and Use Committee of Yeungnam University (AEC2015-006).

### 2.8. Immunocytochemistry

Immunocytochemistry was performed using antibodies against PAX7, MYOD, MYH, IgLON4, IgLON5, NCAM, and CDH15. In brief, C2C12 cells were grown on glass-bottomed dishes, washed twice with PBS, and fixed with 4% formaldehyde for 10 min. After permeabilization with 0.2% Triton X-100 (Sigma Aldrich, St. Louis, MO, USA), cells were incubated overnight at 4 °C in a humid environment with PAX7, MYOD, IgLON4, MYH, NCAM, or CDH15 primary antibodies (1:50, Santa Cruz Biotechnology, Santa Cruz, CA, USA), or IgLON5 primary antibody (1:250, Bioss Antibodies, Woburn, MA, USA). Secondary antibodies (1:100, Alexa Fluor 594 goat antirabbit or mouse antibody SFX kit; Molecular Probes, Invitrogen, Carlsbad, CA, USA) were applied for 1 h at room temperature in the dark. After rinsing cells twice with PBS, nuclei, F-actin (a cytoskeleton marker), or lipid rafts were counterstained with DAPI (1:1000; Sigma Aldrich, St. Louis, MO, USA), phalloidin (1:400, Invitrogen, Carlsbad, CA, USA), and cholera toxin subunit B (1:2000, Thermo Fisher Scientific, Waltham, MA, USA). Fluorescence imaging was performed as previously described [14].

### 2.9. Fusion Indices

On DD4, C2C12 cells were stained with MYH, as previously described [14]. Fusion indices were then calculated.

### 2.10. Total RNA Extraction, cDNA Synthesis, and Real-Time RT-PCR

Total RNA was extracted from cells using Trizol^®^ reagent (Invitrogen, Carlsbad, CA, USA), according to the manufacturer’s instructions, and cDNA was synthesized using a high-capacity cDNA reverse transcription kit (Applied Biosystems, Foster City, CA, USA). Real-time RT-PCR was performed using Power SYBR Green PCR Master Mix (Applied Biosystems, Foster City, CA, USA) as previously described [14]. The primers used are detailed in Supplementary Table S2.

### 2.11. Extraction of Lipid Rafts

Cells were collected before differentiation on DD2 by trypsinization and centrifuged at 1000× *g* for 3 min. The supernatant was then removed; pellets were washed with PBS and centrifuged at 1000× *g* for 3 min. Lipid rafts were extracted from the cell pellets using a spin-column and a nonionic detergent containing buffer provided in the Minute™ Plasma Membrane-Derived Lipid Raft Isolation Kit (Invent Biotechnologies, Plymouth, MN, USA). Proteins in lipid rafts were analyzed by Western blot analysis.

### 2.12. Western Blot Analysis

C2C12 cells or muscle tissues were lysed using RIPA buffer containing a 1% protease inhibitor cocktail (Thermo Fisher Scientific, Waltham, MA, USA), and total protein concentrations were quantified using the Bradford assay. Proteins (50 μg) were electrophoresed in 8% or 10% SDS-PAGE and transferred to PVDF membranes (Millipore, Billerica, MA, USA), which were then blocked with 3% skim milk or BSA (Bovine serum albumin) in TBS (Tris-buffered saline) containing 0.1% Tween 20 for 1 h and incubated with protein-specific primary antibodies [IgLON4 (1:400), IgLON5 (1:1000), PAX7 (1:400), MYOD (1:400), MYOG (1:400), MYH (1:400), NCAM (1:400), CDH15 (1:400), WASP (1:400), CAV1 (1:400), CAV3 (1:400), FLOT-1 (1:400), and ꞵ-actin (1:2000)] in TBS containing 1% skim milk or BSA overnight at 4 °C. Blots were washed and incubated with horseradish peroxidase-conjugated secondary antibodies (goat antimouse or antirabbit; Santa Cruz Biotechnology, Santa Cruz, CA, USA) at room temperature for 2 h and then reacted with Super Signal West Pico Chemiluminescent Substrate (Thermo Fisher Scientific, Waltham, MA, USA). Band chemiluminescence was observed using an Azure 300 chemiluminescent imager (Azure Biosystems, Dublin, CA, USA).

### 2.13. Immunohistochemistry and Immunofluorescence

Paraffin-embedded gas muscle tissues (non-injected or CTX-injected) were deparaffinized with xylene (Junsei, Tokyo, Japan), rehydrated using an ethanol series (Millipore, Billerica, MA, USA), and treated with 0.3% H_2_O_2_/methanol to block endogenous peroxidase activity. Sections were either stained with H&E (hematoxylin and eosin) or blocked with 1% normal goat serum (Thermo Fisher Scientific, Waltham, MA, USA), incubated with IgLON4 antibodies (1:50) overnight at 4 °C, and then with horseradish peroxidase-conjugated secondary antibody (1:100). Sections were stained by adding diamino-benzidine and hydrogen peroxide and observed under a light microscope (Leica, Wetzlar, Germany).

For the immunofluorescence study, gas tissues were deparaffinized and hydrated (as described above) and blocked using 1% goat serum. Sections were then incubated with IgLON4 (Santa Cruz Biotechnology, Santa Cruz, CA, USA) and laminin antibodies (1:50) at 4 °C overnight and then with secondary antibody (1:100) from the Alexa Fluor 594 goat mouse antibody SFX kit. The sections were then stained with (diamino-benzidine and hydrogen peroxide) DAPI, phalloidin, and laminin to counterstain nuclei and cytoskeletons. Fluorescence images were captured in the same manner as in immunocytochemistry.

### 2.14. Statistical Analysis

The significance of differences between mean normalized expressions were determined using Tukey’s test. Real-time RT-PCR, adhesion, proliferation, and fusion indices were analyzed by one-way ANOVA using PROC GLM in SAS ver. 9.0 (SAS Institute, Cary, NC, USA). Statistical significance was accepted for *p* values <0.05.

## 3. Results

### 3.1. Expressions of IgLONs in Mouse Muscles

#### 3.1.1. Expressions of IgLONs in C2C12 Cells and Primary MSCs

**Figure 1:**
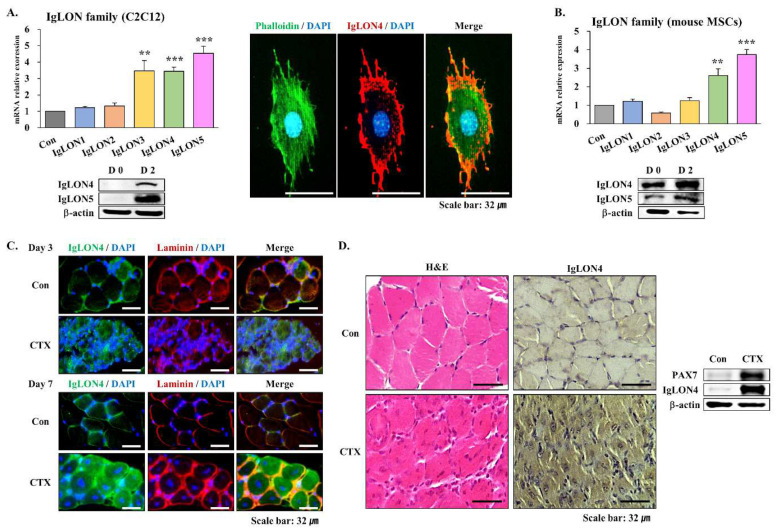
IgLON expressions in murine C2C12 myoblasts and primary MSCs during myogenic differentiation, and IgLON4 protein expression in regenerating mouse muscle: (**A**) mRNA and protein expressions of IgLONs (IgLON1 to 5) in C2C12 myoblasts on differentiation day 2 (DD2) as determined by real-time RT-PCR, and protein expressions of IgLON4 and 5 on DD2 as determined by Western blot. Locations of IgLON4 protein were determined by immunocytochemistry. (**B**) mRNA expressions of IgLONs in mouse primary MSCs on DD2 as determined by real-time RT-PCR, and protein expressions of IgLON4 and 5 as determined by Western blot analysis. (**C**) Expressions of IgLON4 in control and CTX-injected mouse gastrocnemius muscles during regeneration (differentiation days 3 and 7), as determined by immunofluorescence; muscle was collected and embedded with paraffin and then stained with IgLON4 and laminin (Green: IgLON4, Red: Laminin, Blue: Nucleus). (**D**) Expressions of IgLON4 in control and CTX-injected mouse gastrocnemius muscles during regeneration (differentiation day 7), as determined by H&E staining and immunohistochemistry. Protein expressions of PAX7 and IgLON4 were determined by Western blot analysis. Real-time PCR results were normalized to each control (con, before differentiation) and analyzed by *t*-test. Means ± SD (*n* ≥ 3). * *p* < 0.05, ** *p* < 0.01, *** *p* < 0.001.

C2C12 cells (murine myoblasts) and primary MSCs were used to study the expressions of IgLON genes in muscle. In C2C12 cells, the mRNA expressions of IgLON3, 4, and 5 and the protein expressions of IgLON4 and 5 were elevated in the early myogenic differentiation stage (differentiation day 2 (DD2)) versus baseline. However, the mRNA expressions of IgLON1 and 2 mRNA were unaltered after differentiation. IgLON4 protein was mostly expressed on cell membranes, as evidenced by phalloidin (filamentous actin) counterstaining (Figure 1A). Similarly, the mRNA and protein expressions of IgLON4 and 5 were increased on DD2 in mouse primary MSCs, but the mRNAs of IgLON1, 2, and 3 were unaltered (Figure 1B). The extraction purity of mouse MSCs was confirmed using PAX7 (paired box protein Pax-7; an MSC marker) and MYOD (an initial myogenic differentiation marker), which revealed positivities of > 90% and ≥ 80%, respectively (Figure S1).

#### 3.1.2. Muscle Regeneration

To investigate the role of IgLON4 in muscle regeneration, IgLON4 protein expression in CTX-injected mouse muscle was compared to that in control muscle (PBS-injected muscle). Laminin counterstaining at different time points (DD3 and 7) showed that IgLON4 was localized near cell membranes in control mouse muscles but was distributed throughout cells in CTX-injected mouse muscles (Figure 1C). On the seventh day after CTX injection, nuclei and cytoplasms were observed by hematoxylin and eosin (H&E) staining. In normal muscle tissue, nuclei were normally located toward the edge of the cytoplasm. However, the nuclei of CTX-injected muscle cells were located centrally in the cytoplasm, which is typically observed in regenerating muscle. In addition, a significant number of immune cells were seen around myofibers. IgLON4 protein expression in CTX-injected muscles was higher than in control muscles. Immunofluorescence was used to locate IgLON4 protein in tissues. Furthermore, Western blot analysis revealed that IgLON4 and PAX7 protein levels had increased in CTX-injected muscles (Figure 1D). These results show that IgLON4 is localized near the cell membrane in normal cells but throughout cytoplasm during muscle regeneration.

### 3.2. The Function of IgLON4 during Myoblast Differentiation

**Figure 2:**
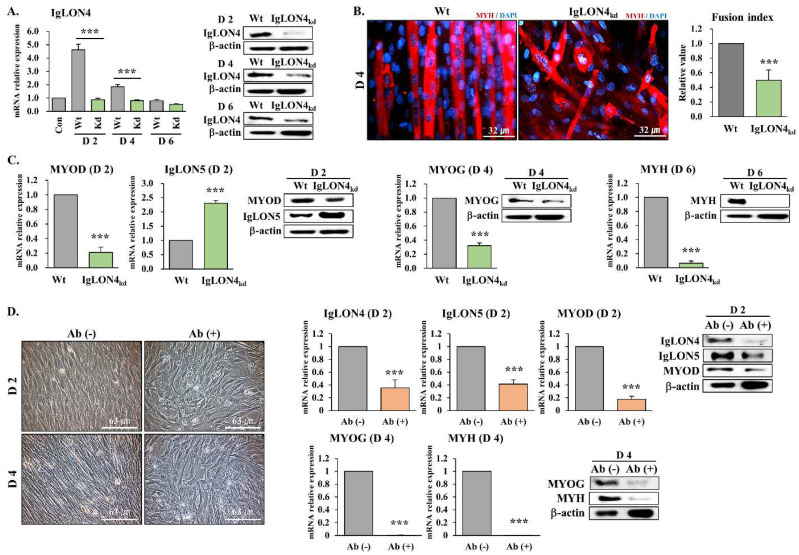
Inhibitory effect of IgLON4 gene and protein expression on C2C12 myoblast differentiation: (**A**) mRNA and protein expressions of IgLON4 as determined by real-time RT-PCR and Western blot analysis in Wt and IgLON4_kd_ C2C12 myoblasts on differentiation days (DDs) 2, 4, and 6. mRNA levels on DDs 2, 4 and 6 were normalized versus Wt levels. (**B**) Expression of MYH by immunocytochemistry in Wt and IgLON4_kd_ C2C12 myoblasts on DD4, and IgLON4_kd_ fusion indices. (**C**) mRNA and protein levels of MYOD, IgLON5, MYOG, and MYH as determined by real-time RT-PCR and Western blot analysis in Wt and IgLON4_kd_ cells on DDs 2, 4, and 6. mRNA and protein levels were compared at each time point. (**D**) Morphologies of cells treated with or without IgLON4 antibody on DD2 or 4. mRNA and protein expressions of IgLON4, IgLON5, MYOD, MYOG, and MYH in cells treated with or without IgLON4 antibody on DDs 2 or 4. mRNA and protein levels at each time-point were compared. Wt indicates transfection with scrambled vector. Real-time PCR results were normalized to each control (con, before differentiation) and analyzed by t-test. Means ± SDs (*n* ≥ 3). * *p* < 0.05, ** *p* < 0.01, *** *p* < 0.001.

IgLON4 gene knockdown (kd) was used to determine the effect of IgLON4 on muscle differentiation. IgLON4 mRNA and protein expressions in the IgLON4_kd_ C2C12 cells were significantly reduced on DD2, 4, and 6 (Figure 2A). Immunocytochemistry using a specific antibody for MYH and fusion indices revealed that myotube formation by IgLON4_kd_ myoblasts was reduced as compared with the wild type (Wt) controls (Figure 2B). Furthermore, when comparing Wt and IgLON4_kd_ for each myogenic marker based on their peak time point, both the mRNA and protein levels of MYOD (DD2), MYOG (DD4), and MYH (DD6) were significantly lower in IgLON4_kd_ cells than in Wt cells, but IgLON5 mRNA and protein levels were significantly higher on DD2 (Figure 2C). When IgLON4 was suppressed, the expression of IgLON5 was increased by a compensatory mechanism.

We examined the effect of blocking IgLON4 protein directly on cell membrane surfaces using an IgLON4-specific antibody. During the first two days of differentiation, normal C2C12 myoblasts proliferate, migrate, and align, and on the fourth day, fuse to form myotubes. However, when cells were treated with IgLON4-specific antibody, alignments were distorted, and cells did not fuse on day 4. As was observed for IgLON4 gene knockdown, IgLON4 protein inhibition resulted in reduced mRNA and protein levels of MYOD (DD2), MYOG (DD4), and MYH (DD4); IgLON4 and IgLON5 mRNA and protein expressions were also reduced (Figure 2D). Interestingly, IgLON5 protein inhibition and IgLON4 knockdown had opposite effects on the expressions of myomarkers (Figure 2C). These findings show that blocking the IgLON4 gene or protein affects myotube formation and the underlying expressions of myogenic markers.

### 3.3. IgLON4 Expression during Myoblast Differentiation, Adhesion, and Proliferation

#### 3.3.1. Effect of IgLON4 and IgLON5 Double Knockdown on Myoblast Differentiation

**Figure 3:**
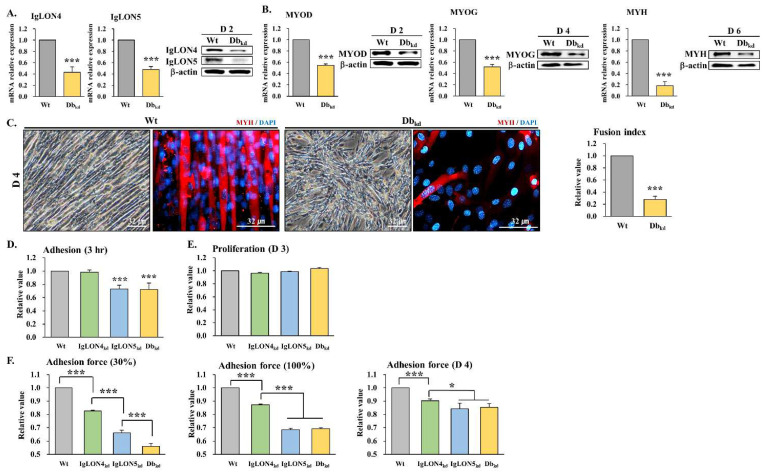
Differentiation, adhesion, and proliferation of Wt, IgLON4_kd_, IgLON5_kd_, and Db_kd_ C2C12 myoblasts: (**A**) The mRNA and protein expressions of IgLON4 and IgLON5 in Wt and Db_kd_ C2C12 myoblasts on differentiation day 2 (DD2) as determined by real-time RT-PCR and Western blot. (**B**) mRNA and protein expressions of MYOD, MYOG, and MYH in Wt and Db_kd_ cells on DDs 2, 4, or 6 as determined by real-time RT-PCR and Western blot analysis. mRNA and protein levels were compared at each time point. (**C**) Wt and Db_kd_ C2C12 myoblast morphologies, MYH expressions (as determined by immunocytochemistry), and fusion indices of Db_kd_ cells on DD4. (**D**, **E**) MTS assays were used to assess Wt, IgLON4_kd_, IgLON5_kd_, and Db_kd_ C2C12 myoblast cell adhesions and proliferation after differentiation for 3 h or 3 days, respectively. (**F**) Wt, IgLON4_kd_, IgLON5_kd_, and Db_kd_ cells were cultured until 30% or 100% confluent, or for 4 days. Plates were then sealed and placed inverted and centrifuged using a swinging bucket rotor at 100×, 500×, or 1000× *g*. Relative cell counts after centrifugation, as assessed by MTS assay and normalized versus initial cell numbers (before centrifugation). Wt indicates transfection with a scrambled vector. Real-time PCR results were normalized to each control (con, before differentiation) and analyzed by t-test. Means ± SDs (*n* ≥ 3). * *p* < 0.05, ** *p* < 0.01, *** *p* < 0.001.

Since IgLON family members have adhesion-related functions, we investigated the effect of IgLON4_kd_ on myoblast adhesion. Double knockdown (Db_kd_) of IgLON4 and 5 was performed to avoid possible interference by the compensatory increase in IgLON5 expression after IgLON4_kd_. The effectiveness of Db_kd_ was confirmed by assessing the expressions of IgLON4 and 5 at the gene and protein levels on DD2. Both were found to be significantly decreased (Figure 3A). Similarly, the mRNA and protein expressions of MYOD, MYOG, and MYH were markedly lower in Db_kd_ myoblasts than in the Wt (Figure 3B).

Notably, Db_kd_ resulted in a scattered cell morphology, which was reminiscent of the effect of immunoneutralization using IgLON4-specific antibody. The morphological results of Db_kd_ myoblast differentiation indicated that IgLON5 and IgLON4 have distinctive characteristics, with IgLON5 being essential for cell adhesion and IgLON4 presumably being associated with cell arrangement. Moreover, because IgLON4 and IgLON5 had unique effects on cell shape, we hypothesized that they play unique roles. In addition, immunocytochemistry for MYH showed that myotube formation was dramatically inhibited in Db_kd_, and this was supported by fusion indices, which were lower for Db_kd_ than the Wt (Figure 3C).

#### 3.3.2. Effects of IgLON4_kd_, IgLON5_kd_, and Db_kd_ on Myoblast Adhesion, Proliferation, and Adhesive Force

To investigate the functions of IgLON4 more specifically, we compared adhesions, adhesive forces, and the proliferation of Wt, IgLON4_kd_, IgLON5_kd_, and Db_kd_ myoblasts. In a previous study, the adhesion ratio of IgLON5_kd_ to cell culture dishes was lower than that of the Wt [14]. However, we found no significant difference between IgLON4_kd_ and Wt, and the adhesion ratio of Db_kd_ was similar to that of IgLON5_kd_ (Figure 3D and Figure S2A). These results indicate that IgLON5, a cell membrane adhesive factor, contributes to cell adhesion, similar to what we previously demonstrated [14], whereas IgLON4 does not. The comparable adhesion decreases exhibited by Db_kd_ and IgLON5_kd_ indicated that the decrease in adhesion ratio shown by Db_kd_ was due to IgLON5_kd_ rather than IgLON4_kd_. No significant difference in cell proliferation was observed for 3 days based on adhesive cells of Wt, IgLON4_kd_, IgLON5_kd_, or Db_kd_ (Figure 3E and Figure S2B). Cell-to-cell and cell-to-culture dish detachments were assessed using centrifugal *g* forces for Wt, IgLON4_kd_, IgLON5_kd_, and Db_kd_ myoblasts. When cells had reached ~30% confluence of the cell culture dish, they were centrifuged at 100× *g* to check for detachment. We found that cell adherence followed the order Wt > IgLON4_kd_ > IgLON5_kd_ > Db_kd_. When cells were centrifuged at 500× *g* after reaching 100% confluence, IgLON5_kd_ and Db_kd_ detached most, followed by IgLON4_kd_. Furthermore, on DD4, centrifugation at 1000× *g* was performed to compare adhesive strengths during myogenic differentiation, and the results obtained followed the order Wt > IgLON4_kd_ > IgLON5_kd_ > Db_kd_ (Figure 3F). Thus, in terms of cell adhesion, IgLON4 and IgLON5 appeared to function similarly, but not as potently as IgLON5. When cells were examined under a microscope after centrifugation, Db_kd_ was found to have adhered the least, followed by IgLON5_kd_ (Figure S3). These findings suggest that both IgLON4 and 5 have no effect on proliferation but that the absence of IgLON4 and 5 results in a significant decrease in myoblast adhesion, which indicates that IgLON5, rather than IgLON4, is responsible for cell adhesion.

### 3.4. Participation of IgLON4 in the Regulation of Myoblast Differentiation Involves Cell-to-Cell Adhesion and Alignment Functions

**Figure 4:**
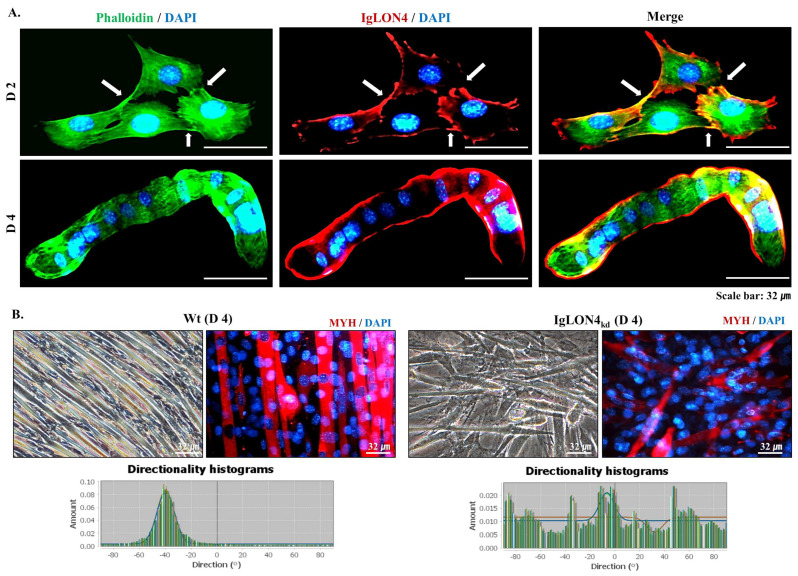
IgLON4 protein expression and myotube orientation in Wt and IgLON4_kd_ C2C12 myoblasts during differentiation: (**A**) IgLON4 protein locations were determined by immunocytochemistry on differentiation days (DDs) 2 and 4. Phalloidin and IgLON4 were fluorescently labeled green and red, respectively. Phalloidin was used for counterstaining cytoskeletal actin filaments. (**B**) Myotube formation was observed by MYH immunocytochemistry in Wt and IgLON4_kd_ cells on DD4. Directional analysis of myotube formation by Wt and IgLON4_kd_ cells was performed using ImageJ on DD4. Wt indicates transfection with a scrambled vector.

When the location of IgLON4 expression during myogenic differentiation was observed in detail, we found it was centered at or near sites of cell-to-cell contact. These observations were best observed on DD2 when cell adhesion and alignment peaked. In addition, we observed IgLON4 in cell membranes during cell fusion and the formation of multinuclear myotubes (DD4) (Figure 4A). These results are consistent with the protein expression pattern of IgLON4, which was strongly expressed in the myofiber membranes of normal mice (Figure 1C). Furthermore, high IgLON4 expression was noticed during muscle differentiation, and IgLON4 expression was concentrated at sites of cell-to-cell binding (Figure S4). These observations indicate that IgLON4 participates in the formation of a framework that facilitates the differentiation of surrounding cells and serves as a myotube alignment reference.

We also investigated the morphologies of myotubes produced by IgLON4_kd_ and Wt myoblast differentiation. In the case of the Wt, uniformly arranged striated myotubes characteristic of SMs were observed, however; in the case of IgLON4_kd_, myotubes were distorted and irregularly arranged. MYH staining confirmed these observations. In addition, myotube alignments after myogenic differentiation of Wt and IgLON4_kd_ myoblasts were studied using ImageJ, which revealed that, unlike Wt myotubes, which converged in one direction, IgLON4_kd_ myotubes were randomly oriented (Figure 4B). These findings suggest IgLON4 is necessary for the uniform arrangement and orientation of myotubes.

### 3.5. The Effect of IgLON4 on Muscle Differentiation Involved Lipid Raft Accumulation

**Figure 5:**
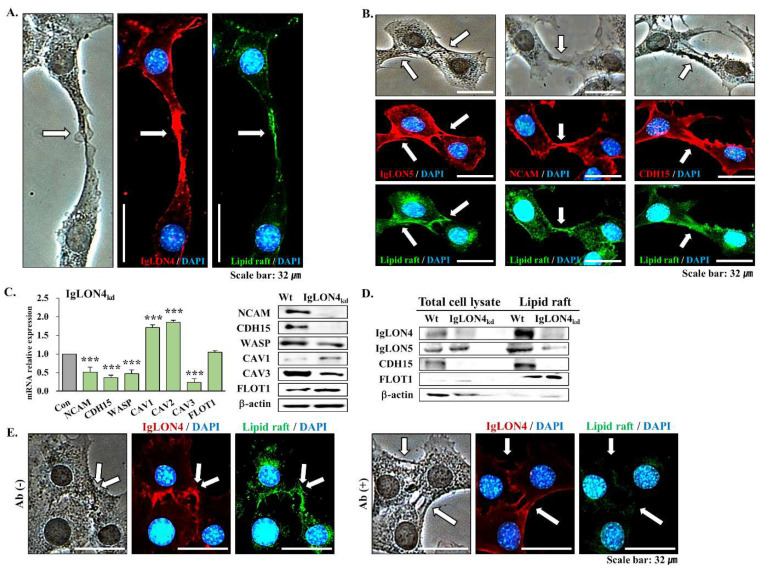
Locations of IgLON4 protein and lipid rafts on membranes, and the inhibitory effects of IgLON4 mRNA and protein on lipid raft during myoblast differentiation: (**A**) Locations of IgLON4 protein and lipid rafts as determined by immunocytochemistry on DD2. (**B**) Locations of IgLON5, NCAM, and CDH15 proteins and lipid rafts as determined by immunocytochemistry on DD2. (**C**) mRNA and protein expressions of NCAM, CDH15, WASP, CAV1, CAV2, CAV3, and FLOT1 in Wt and IgLON4_kd_ C2C12 myoblasts on DD2 as determined by real-time RT-PCR and Western blot analysis. (**D**) Extraction of lipid rafts from Wt and IgLON4_kd_ C2C12 myoblasts on DD2, and the protein expressions of IgLON4, IgLON5, CDH15, and FLOT1 by Western blot analysis. FLOT1 and β-actin were used as markers of lipid rafts and total cell lysates, respectively. (**E**) Locations of IgLON4 protein and lipid rafts in C2C12 myoblasts treated with or without IgLON4 antibody as determined by immunocytochemistry on DD2. Wt indicates transfection with a scrambled vector. Lipid rafts were labeled green using cholera toxin and IgLON4, IgLON5, NCAM, and CDH15 were labeled red using Alexa Fluor 594. Real-time PCR results were normalized to each control (con, before differentiation) and analyzed by t-test. Means ± SDs (*n* ≥ 3). * *p* < 0.05, ** *p* < 0.01, *** *p* < 0.001.

IgLON4 has been reported to cluster in the lipid rafts of rat brain cell membranes [22]. We explored the association between IgLON4 and lipid rafts during myogenic differentiation and focused on the distributions of IgLON4 and lipid rafts where cells meet during the differentiation process. IgLON4 was highly expressed in junctions between cells, and lipid rafts were observed in overlapping IgLON4 protein-expressed regions (Figure 5A). These findings show that IgLON4 and lipid rafts accumulate where myoblasts interact during the early differentiation stage.

**Figure 6:**
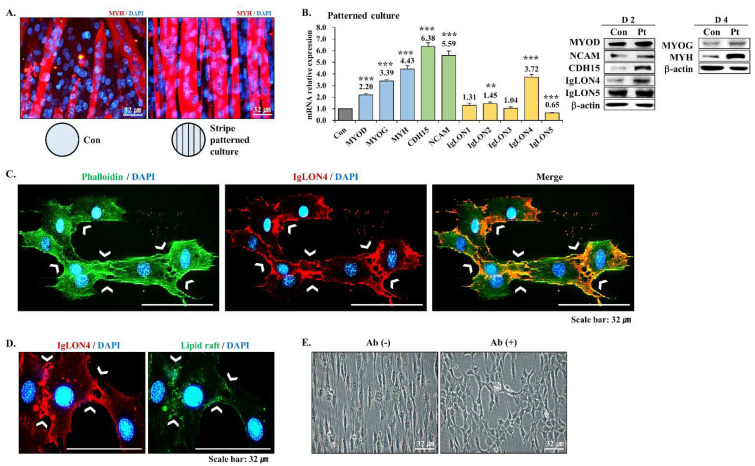
Effect of using striped culture plates on C2C12 myoblasts differentiation: (**A**) The formation of myotubes in normal and striped plate cultures was observed by MYH immunocytochemistry. (**B**) mRNA and protein expressions of myogenic markers, muscle-specific adhesion proteins, and IgLON family members in C2C12 myoblasts cultured in normal and striped plates on DD2 and 4 as determined by real-time RT-PCR and Western blot analysis. (**C**) Location of IgLON4 protein as determined by immunocytochemistry on DD2 in C2C12 myoblasts cultured on striped plates. Labeling was performed using fluorescently labeled phalloidin and IgLON4 (green and red, respectively). Phalloidin was used to counterstain cytoskeletal actin filaments. (**D**) Locations of lipid rafts and IgLON4 protein as determined by immunocytochemistry on DD2. Lipid rafts were stained using labeled cholera toxin (green fluorescence) and IgLON4 with Alexa Fluor 594 (red fluorescence). (**E**) Morphologies of C2C12 myoblasts treated with or without IgLON4 antibody on DD2. Real-time PCR results were normalized to each control (con, before differentiation) and analyzed by t-test. Means ± SD (*n* ≥ 3). * *p* < 0.05, ** *p* < 0.01, *** *p* < 0.001.

The locations of IgLON5, neural cell adhesion molecule (NCAM), and cadherin 15 (CDH15), which are known to be associated with adhesion during muscle development, were compared with the locations of lipid rafts to confirm the relationship between lipid rafts and muscle differentiation. IgLON5, NCAM, and CDH15 were highly expressed where myoblasts contacted each other (Figure 6B white arrow), that is, where myoblasts congregated, and lipid rafts also accumulated in these regions (Figure 5B).

Muscle-specific adhesion (NCAM and CDH15) and fusion- (Wiskott–Aldrich syndrome protein, WASP) related gene and protein expressions were reduced during the myogenic differentiation of IgLON4_kd_ myoblasts. To confirm the effect of IgLON4_kd_ on lipid rafts, we examined the gene and protein expression patterns of caveolin 1 to 3 (CAV 1 to 3), which are well-known lipid raft markers [23]. The majority of tissue cells contained CAV1 and CAV2 [24]. During IgLON4_kd_ differentiation, CAV1 and 2 gene and protein levels were significantly higher than in the Wt; however, CAV3, which is specific for SM [25], exhibited significant reductions at the gene and protein levels. Furthermore, no significant changes were found in the expression of Flotillin 1 (FLOT1, a lipid raft-constituting factor) at the gene or protein levels (Figure 5C). Consequently, myogenic differentiation in the absence of IgLON4 was characterized by reductions in SM-specific adhesion and fusion factors, increases in the non-muscle-specific lipid raft markers (CAV1 and CAV2), and decreases in muscle-specific lipid rafts (CAV3), indicating that IgLON4_kd_ alters the expressions of essential lipid raft proteins.

To confirm the presence of IgLON4 in lipid rafts and its effects on myogenic differentiation, lipid rafts were extracted from Wt and IgLON4_kd_ myoblasts on DD2. IgLON4 protein levels in lipid rafts extracted from Wt cells were much higher than in total cell lysates, whereas IgLON4_kd_ cells in total lysates and extracted lipid rafts showed decreased IgLON4 protein levels. Similar to IgLON4, IgLON5 was found to have elevated protein expression in the extracted lipid raft. In the case of IgLON4_kd_ cells, IgLON5 expression in total cell lysates was higher than in the Wt but lower than in the extracted lipid rafts. CDH15 is a muscle adhesion factor expressed in lipid rafts [26]. We found that CDH15 protein levels were higher in extracted lipid rafts than in total cell lysates in Wt cells, and that its expression in IgLON4_kd_ myoblasts was significantly lower in total cell lysates and extracted lipid rafts than in Wt cells. The expression of FLOT1, which is abundant in lipid rafts, was weak in the total cell lysates of Wt and IgLON4_kd_ myoblasts, but a clear band was observed for extracted lipid rafts and in Wt and IgLON4_kd_. A ꞵ-actin band was observed in the total cell lysates of Wt and IgLON4_kd_ cells, but not in all extracted lipid rafts (Figure 5D). These findings show that IgLON4, IgLON5, and CDH15 levels were higher in lipid rafts compared to total cell lysates in the differentiation of Wt cells However, in the case of IgLON4_kd_ cells, both total cell lysates and lipid raft were decreased.

Immunoneutralization with an IgLON4-specific antibody was used to observe these effects under a microscope. IgLON4 was strongly expressed, and lipid rafts accumulated in the cell-to-cell junctions of myoblasts not treated with IgLON4 antibody on DD2. However, in myoblasts treated with IgLON4 antibody, both IgLON4 expression and lipid raft accumulation was reduced (Figure 5E).

### 3.6. Effect of an Aligned Environment on Myogenic Differentiation

We found that IgLON4 not only regulates myoblast alignment to facilitate fusion during myogenic differentiation but also contributes to muscle differentiation by accumulating in lipid rafts between cells. When myogenic differentiation was analyzed in striped cell culture dishes, myotube shapes were straighter and better aligned than in normal cell culture dishes (Figure 6A). In addition, MYOD, MYOG, and MYH, and CDH15 and NCAM (both attachment factors that promote muscle growth) gene and protein levels were also greater in striped dishes. Furthermore, IgLON4 expression increased most among IgLONs, followed by IgLON2 expression, and IgLON5 expression decreased in striped dishes (Figure 6B). These results show that a well-aligned environment promotes muscle differentiation and suggest that IgLON4 and an aligned environment promote myoblast alignment (Figure 6C). Furthermore, lipid rafts accumulated at cell attachments, and these accumulations overlapped with IgLON4 expression (Figure 6D). Finally, we examined the changes that occurred in this aligned environment after blocking IgLON4 protein function by immunoneutralization. On DD2, myoblasts were well organized in striped dishes. However, when IgLON4 antibody was added, cell shapes and alignments and cell-to-cell adhesion patterns were disrupted (Figure 6E). These observations confirmed that IgLON4 plays a crucial role in cell alignment and the establishment of an aligned, adherent morphology during myoblast differentiation.

## 4. Discussion

Various ECM components, membrane proteins, and transmembrane receptors, such as FMOD [6,7], MGP [9], IgLON5 [14], and DPT [12], play important roles in the regulation of myogenesis [4]. The roles of IgLON5 in myogenesis and muscle regeneration were recently investigated in mice [14], and in an FMOD_kd_ microarray study, IgLON5 was significantly downregulated during muscle development [7]. In this study, IgLON4, which has been linked to neurodevelopmental processes, was investigated in the context of SM [18]. Little information is available on IgLON4, particularly in the SM setting, or on its effects on lipid rafts. Here, we investigated the effects of IgLON4 on SM myogenesis, and lipid rafts and their influence on myoblast adhesion and orientation.

Several authors have reported that IgLONs are the most abundant GPI-anchored proteins expressed in neurons [27,28]. IgLONs are considered to play key roles throughout the life cycle because they are expressed early during embryogenesis and persist in maturity. Furthermore, they have been linked to a range of diseases [29]. Almost all members of the IgLON family are expressed in neurons and oligodendrocytes; IgLON2 is the exception and is expressed exclusively in neurons [30,31]. IgLON1, 2, 3, and 4 are proteins that facilitate neurite extension and synaptogenesis [31,32], and IgLON4 is also expressed in the digestive, genitourinary, and respiratory systems and in the sensory organs of mice [33].

We investigated the expression of IgLONs in mice and found that only IgLONs 4 and 5 were significantly expressed and were predominantly localized to the membranes. Furthermore, IgLON4 was overexpressed in CTX-injected mouse muscles, suggesting its involvement in muscle regeneration (Figure 1). IgLON4 appears to work in the same way as IgLON5, which was recently reported to be involved in muscle regeneration [14].

Furthermore, the knockdown of IgLON4 at the gene and protein levels suppressed myotube formation and the underlying expressions of myogenic markers, suggesting that it plays an important role in muscle development. Observed increases in the expression of IgLON5 genes and proteins during the differentiation of IgLON4 _kd_ cells were considered to be the result of a compensatory effect among IgLON family members. IgLONs exhibit significant intrafamily homology, and functional compensation has been observed between other IgLON genes. According to Vanaveski et al., IgLON adhesion molecules serve a wide range of purposes, and the loss of one family member is not compensated for by a quantitative increase in the others. It was also suggested that intrafamily interactions, including potential compensatory effects, might be tissue-specific [31].

The specific role of IgLON4 in myogenic differentiation, adhesion, and proliferation was then investigated. To avoid the possible effects of increased IgLON5 expression after IgLON4 knockdown, double knockdown (Db_kd_) of IgLON4 and 5 was performed. Interesting results were obtained by comparing the morphologies of Db_kd_ and Wt cells during differentiation. Morphologies were altered in the same way as morphologies after immunoneutralization with IgLON4-specific antibody, which indicated IgLON5 and IgLON4 are involved in adhesion and possibly cell arrangement and that the roles of IgLON4 and IgLON5 are distinct. In previous studies, IgLON5_kd_ myoblasts failed to adhere to each other and detached from cell culture dishes during differentiation [14], and we observed the same effect after Db_kd_ (Figure 3A–C).

Furthermore, Db_kd_ had no significant effect on cell proliferation up to DD3, indicating that IgLON4 and 5 do not play a role in proliferation, which is consistent with our previous study on IgLON5 [14] (Figure 3D–F). It has also been suggested that IgLON1 controls astrocyte proliferation and growth [34,35].

We also found that IgLON4 regulated cell-to-cell adhesion and alignment during myoblast differentiation. IgLON4 was highly expressed at cell-to-cell adhesions on DD2 and was consistently expressed in cell membranes throughout differentiation, even during cell fusion (Figure 4A). During MSC differentiation, IgLON4 was expressed at high levels at cell-to-cell adhesion sites. Notably, blocking IgLON4 had a remarkable impact on myotube alignment. This was confirmed by directional analysis, which revealed that IgLON4 most influenced myotube orientation (Figure 4B). It has been demonstrated that changes in fiber orientations in SM tissues alter tissue stiffness [36].

The IgLON family is composed of GPI-anchored proteins, which are predominantly found in lipid rafts, a type of membrane microdomain in which sphingolipids and cholesterol accumulate. Furthermore, IgLON4 predominantly localizes to rafts, especially at cell-to-cell contacts [1], connects to rafts using its GPI-anchor, and is involved in cholesterol transport [37]. During differentiation, we observed that IgLONs (IgLON4 and 5) and adhesion genes (NCAM and CDH15) were abundant at cell junctions and in lipid rafts. CDH15, as a component of the myogenic program, is required for the progression of skeletal myogenesis and may even function as a precursor for terminal muscle differentiation [38].

CAV1, 2, and 3 are membrane proteins with homologous structures. CAV3 is a muscle-specific isoform, and its dysfunction has been linked to various conditions, including diabetes, cancer, atherosclerosis, and cardiovascular disease. Mutations in the CAV3 gene cause caveolinopathies, which are types of muscular dystrophy [39]. CAV3 protein expression was reported to be dramatically elevated during the differentiation of C2C12 myoblasts in vitro [40], and we found CAV3 expression was significantly lower in IgLON4_kd_ myoblasts during differentiation, which indicated that IgLON4_kd_ alters lipid raft composition. Furthermore, blocking IgLON4 protein expressed on cell membrane with IgLON4-specific antibody significantly reduced the accumulation of lipid rafts in cell-to-cell contact areas, implying that IgLON4 promotes muscle differentiation by causing lipid rafts to accumulate.

Interestingly, we also found that an aligned environment promotes the myogenic differentiation of myoblasts. In particular, when we investigated myogenic differentiation in striped cell culture dishes, the myotubes were straighter and more aligned than those cultured in standard culture dishes. These findings suggest that a well-aligned environment promotes muscle differentiation and that IgLON4 promotes myoblast alignment and environmental alignment. In SM, an aligned environment is required for functional muscle regeneration, and aligned biomaterials enhance myotube formation [41]. In the tissue engineering field, an aligned environment is critical for enhancing the myogenic differentiation of myoblasts for therapeutic applications, muscle regeneration, and cultured meat production, and our results suggest that IgLON4 may play key roles in the development of such environments.

## 5. Conclusions

**Figure 7:**
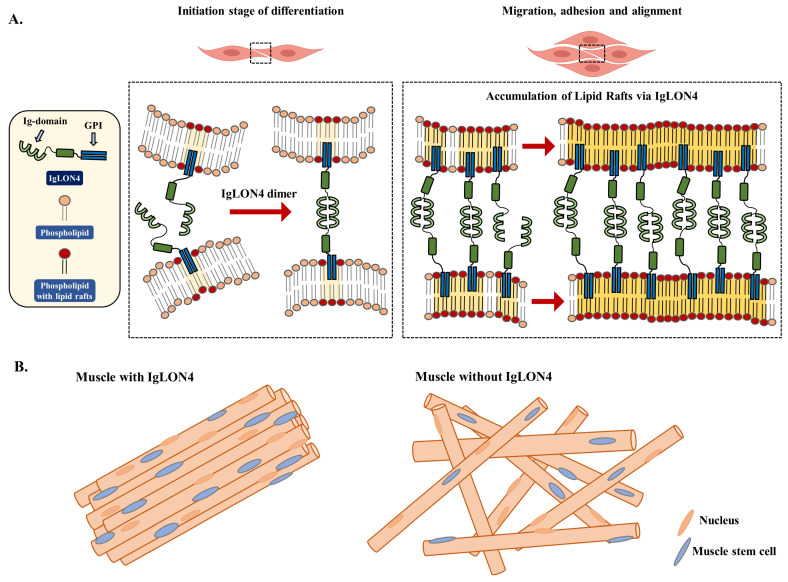
The proposed role of IgLON4 during myoblast differentiation. (**A**) Schematic structure of IgLON4, its membrane location, and its association with lipid raft accumulation during myoblast differentiation (**B**) Illustrations of the hypothetical structure of IgLON4-blocked muscle and normal muscle.

Our findings revealed that IgLON4 has a novel role in SM settings and is involved in (1) cell-to-cell adhesion, (2) myogenesis and regeneration, (3) muscle orientation, and (4) lipid raft accumulation, and (5) is present on membranes and connects cells. Figure 7 provides a graphical illustration of the functions of IgLON4 in SM. IgLON4 has recently been investigated as a therapeutic target in numerous disease settings, including obesity, intellectual disability, schizophrenia, depression, and Alzheimer’s disease [1]. IgLON4 gene knockout mice exhibited substantial increases in fat mass and significant decreases in muscle mass, which suggests IgLON4 ought to be considered a potential molecular target for the development of anti-obesity therapies [20]. The present study contributes to our understanding of the functions of IgLON4 in SM settings, and we hope that it aids the development of therapeutics for muscle mass management.

## Data Availability

Data is contained within the article/Supplementary Files.

## Supplementary Materials

The following supporting information can be downloaded at: https://www.mdpi.com/article/10.3390/cells11203265/s1, Table S1: shRNA construct sequence of IgLON4 and IgLON5. Table S2: Primer sequence of genes. Figure S1. The purity of MSCs isolated from mice was confirmed by immunocytochemistry at baseline and on differentiation day 2, respectively, using the MSC markers PAX7 and MYOD. Ratios of cells expressing each marker were determined by DAPI labeling. Figure S2. Morphology of Wt, IgLON4, IgLON5, and Db_kd_ C2C12 myoblasts adhesion and proliferation: (A) Wt, IgLON4_kd_, IgLON5_kd_, and Db_kd_ C2C12 myoblast adhesions at 3 h after seeding. (B) Wt, IgLON4_kd_, IgLON5_kd_, and Db_kd_ C2C12 myoblast proliferation from 3 days after seeding. Figure S3. Morphologies of Wt, IgLON4_kd_, IgLON5_kd_, and Db_kd_ 30% and 100% confluent C2C12 myoblasts on differentiation day 4. Figure S4. IgLON4 expression in the vicinity of interacting cells.

## Abbreviations

SM	skeletal muscle
MSC	muscle satellite (stem) cells
ECM	extracellular matrix
FMOD	fibromodulin
IgLON	immunoglobulin-like cell adhesion molecule
GPI	glycosylphosphatidylinositol
CAV	caveolin
GAS	gastrocnemius
