# Supplementary material for: IgLON4 Regulates Myogenesis via Promoting Cell Adhesion and Maintaining Myotube Orientation
Source: Cells. 2022 Oct 17;11(20):3265. doi: 10.3390/cells11203265 (PMC9601278; doi:10.3390/cells11203265)
Supplement: Supplementary file 1 [file cells-11-03265-s001.zip › cells-1924875-supplementary.pdf]

IgLON4 regulates myogenesis via promoting cell adhesion and maintaining myotube orientation

Jeong Ho Lim[1,2†], Khurshid Ahmad[1,2†], Hee Jin Chun[1], Ye Chan Hwang[1], Afsha Fatima Qadri[1], Shahid Ali[1], Syed Sayeed Ahmad[1,2], Sibhghatulla Shaikh[1,2], Jungseok Choi[3], Ji Hoi Kim[1,2], Jun-O Jin[1,2], Myung Hee Kim[2,4], Sung Soo Han[2,5], Inho Choi[1,2]*, Eun Ju Lee[1,2]*

Supplementary Tables

Table S1: shRNA construct sequence of IgLON4 and IgLON5

Gene	Catalog #	Hairpin sequence (5′– 3′)	Corresponding shRNA (5′– 3′)
**IgLON4 shRNA Plasmid (m) is a pool of 3 different shRNA plasmids**			
IgLON4	sc-149901-SHA	GATCCCAACAGGTCAAGTATCATTTTCAAGAGAAATGATACTTGACCTGTTGTTTTT	Sense: CAACAGGUCAAGUAUCAUUtt<br>Antisense: AAUGAUACUUGACCUGUUGtt
	sc-149901-SHB	GATCCGTGAACTTTGCGCCTACAATTCAAGAGATTGTAGGCGCAAAGTTCACTTTTT	Sense: GUGAACUUUGCGCCUACAAtt<br>Antisense: UUGUAGGCGCAAAGUUCACtt
	sc-149901-SHC	GATCCCTTCGGCAACTATACTTGTTTCAAGAGAACAAGTATAGTTGCCGAAGTTTTT	Sense: CUUCGGCAACUAUACUUGUtt<br>Antisense: ACAAGUAUAGUUGCCGAAGtt
**IgLON5 shRNA Plasmid (m) is a pool of 3 different shRNA plasmids**			
IgLON5	sc-140623-SHA	GATCCGGAATACGAATGCGTTACTTTCAAGAGAAGTAACGCATTCGTATTCCTTTTT	Sense: GGAAUACGAAUGCGUUACUtt<br>Antisense: AGUAACGCAUUCGUAUUCCtt
	sc-140623-SHB	GATCCCCATCACTGTGAGGGATTATTCAAGAGATAATCCCTCACAGTGATGGTTTTT	Sense: CCAUCACUGUGAGGGAUUAtt<br>Antisense: UAAUCCCUCACAGUGAUGGtt

Table S2: Primer sequence of genes

Mouse	Gene	Product size (bp)	Tm (°C)	Forward primer	Reverse primer
1	**GAPDH**	155	59	5′- TGC TGG TGC TGA GTA TGT CG - 3′	5′- CAA GCA GTT GGT GGT ACA GG - 3′
2	**PAX7**	170	59	5′- GAG TTC GAT TAG CCG AGT GC -3′	5′-CGG GTT CTG ATT CCA CAT CT-3′
3	**MYF5**	105	59	5′- TGA GGG AAC AGG TGG AGA -3′	5′- AGC TGG ACA CGG AGC CCC -3′
4	**MYOD**	213	59	5′- AGG AGC ACG CAC ACT TCT CT - 3′	5′- TCT CGA AGG CCT CAT TCA CT - 3′
5	**MYOG**	185	59	5′- TCC AGT ACA TTG AGC GCC TA - 3′	5′- CAA ATG ATC TCC TGG GTT GG - 3′
6	**MYL2**	177	59	5′- AAA GAG GCT CCA GGT CCA AT - 3′	5′- CCT CTC TGC TTG TGT TGG TCA - 3′
7	**MYH**	141	59	5′- CTG AAG CAG AGG CAA GTA GTG - 3′	5′- CGA AAT GAG GAT GGG TGC TC - 3′
8	**IgLON1**	120	59	5′- GTG CAA GTT CCT CCC CAG ATA- 3′	5′- GTG CCT CCA TGT CAC CGT T - 3′
9	**IgLON2**	157	59	5′- GGT CGA AGG AAA GAA GGG AGT - 3′	5′- ACC GGG ACC AAA TAG CAT GA - 3′
10	**IgLON3**	150	59	5′- TCT TGG CAT TTT GGA ACC AGC - 3′	5′- CTA CCA CAC ACC TGA GGA TGG - 3′
11	**IgLON4**	100	59	5′- AAT GTG ACG CAG GAG CAC TT - 3′	5′- AGG GAA TGG AAC TCT GGT TGA - 3′
12	**IgLON5**	218	59	5′- CTC CGA GAC GGT TTC ACC TC - 3′	5′- ATA GCT TCA CAG CGC AGG AG - 3′
13	**FMOD**	155	59	5′- AGG AGC ACG CAC ACT TCT CT - 3′	5′- TCT CGA AGG CCT CAT TCA CT - 3′
14	**DPT**	224	59	5′- GGA TCG TGA GTG GCA ATT TT - 3′	5′- CGA ATT CGC AGT CGT AGT CA - 3′
15	**COL1a1**	224	59	5′- CTT TGC TTC CCA GAT GTC CT - 3′	5′- CCC CAT CAT CTC CAT TCT TG - 3′
16	**THBS1**	162	59	5′- CAT GTG GCAA TGG AAT TCA G - 3′	5′- AAC AGG ACG ACC ATG GAG AC - 3′
17	**WASP**	201	59	5′- GCT CCA AAT GGT CCC AAT CT - 3′	5′- CCA TCC AAC ATG CCC AAT GTG - 3′
18	**CDH15**	134	59	5′- GGA CTA TGA GAG CCG TGA GC - 3′	5′- GAG CTT CGT TGG TGT CCT GA - 3′
19	**NCAM**	148	59	5′- GTA TGA TGC CAA AGA AGC CAA CA - 3′	5′- GAC TGG CTG TGT CTT GAA CTC - 3′
20	**CAV1**	122	59	5′- CGC ACA CCA AGG AGA TTG AC- 3′	5′- GAT GCC GTC GAA ACT GTG TG- 3′
21	**CAV2**	136	59	5′- TGC AGA GCC TGA GAC TAC AC- 3′	5′- GAT ACC CGC AAT GAA GGC CA- 3′
22	**CAV3**	249	59	5′- TGA TGA CCG AAG AGC ACA CG- 3′	5′- GTG GAA CAC CCA GCA GTG TA- 3′
23	**FLOT1**	101	59	5′- CTC TCT CAA CAC ACT GAC CCT C- 3′	5′- CCT GGA TTT TCA CCT GGG CAA- 3′

Supplementary figures

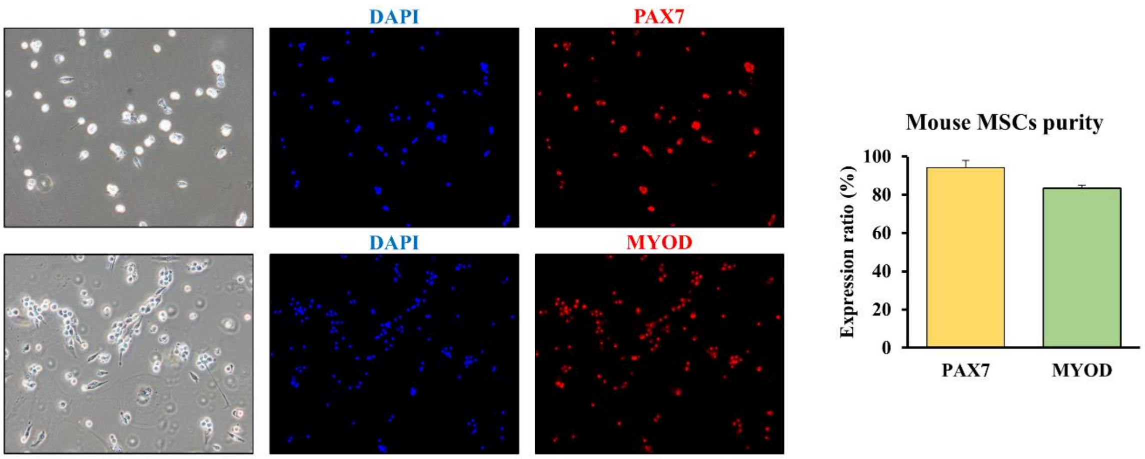

Figure S1. The purity of MSCs isolated from mice was confirmed by immunocytochemistry at baseline and on differentiation day 2, respectively, using the MSC markers PAX7 and MYOD. Ratios of cells expressing each marker were determined by DAPI labeling

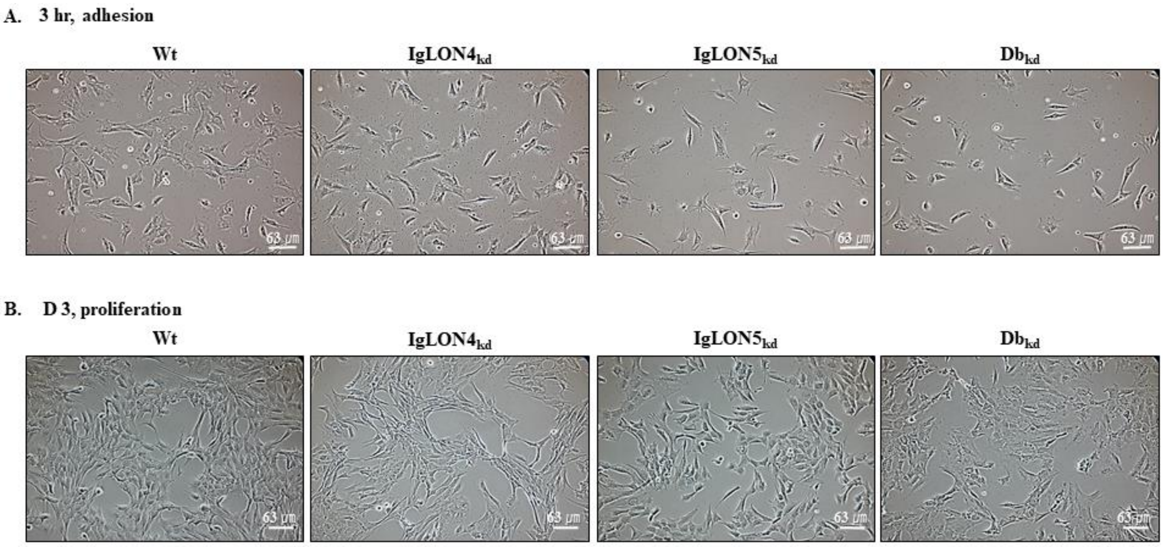

Figure S2. Morphology of Wt, IgLON4, IgLON5, and Db_{kd} C2C12 myoblasts adhesion and proliferation: **A)** Wt, $IgLON4_{kd}$, $IgLON5_{kd}$, and Db_{kd} C2C12 myoblast adhesions at 3 hrs after seeding. **B)** Wt, $IgLON4_{kd}$, $IgLON5_{kd}$, and Db_{kd} C2C12 myoblast proliferations from 3 days after seeding.

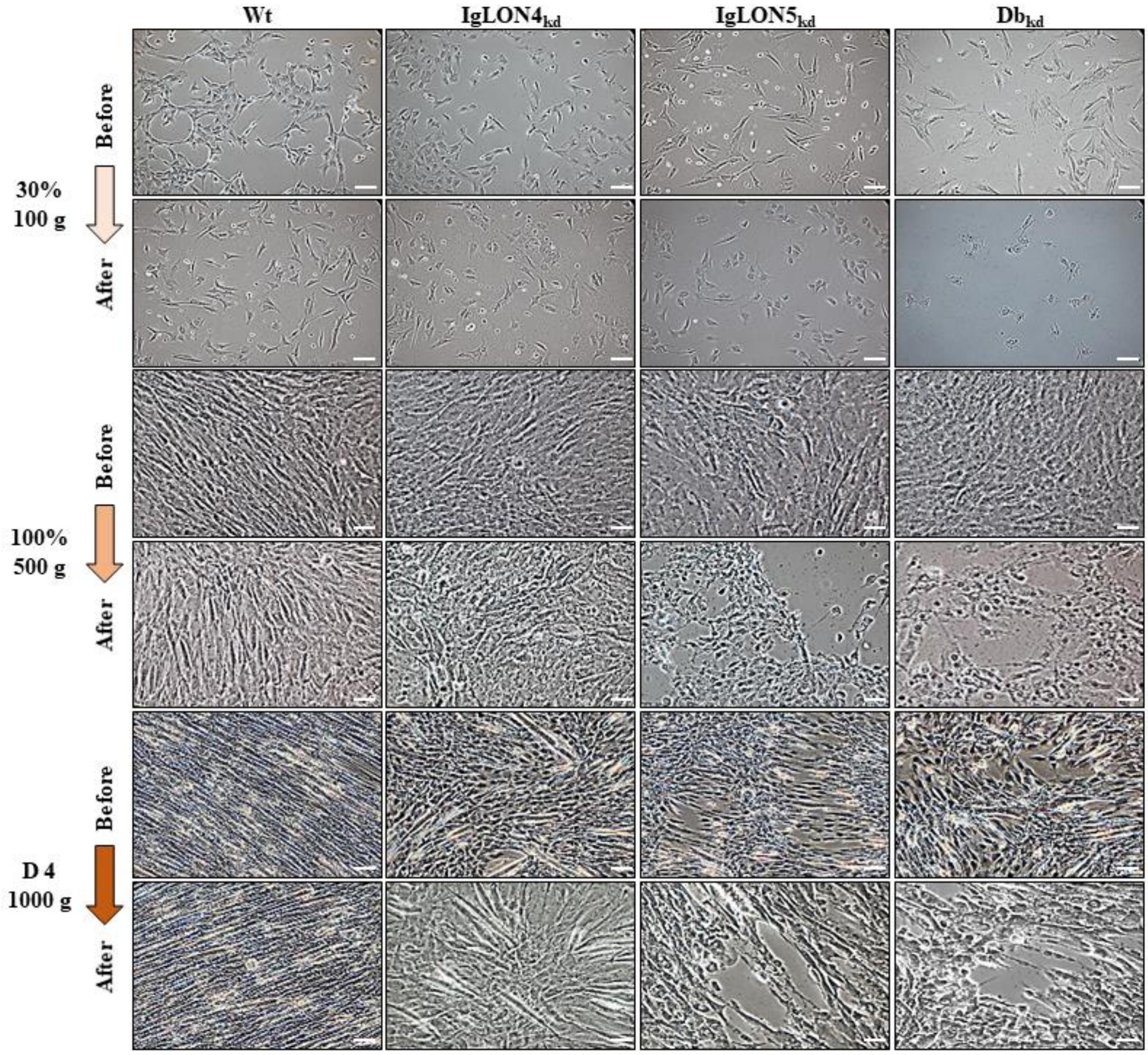

Figure S3. Morphologies of Wt, $IgLON4_{kd}$, $IgLON5_{kd}$, and Db_{kd} 30% and 100% confluent C2C12 myoblasts on differentiation day 4.

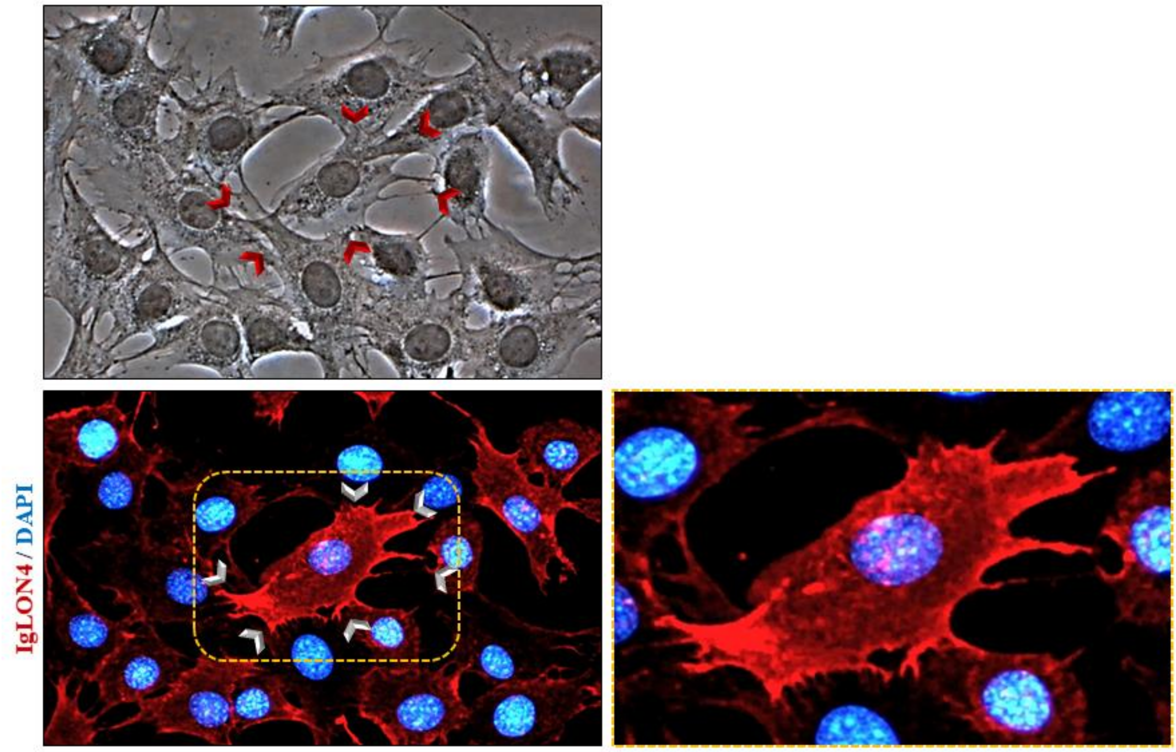

Figure S4. IgLON4 expression in the vicinity of interacting cells.
